# Racial and Ethnic Differences in *BRCA1/2* and Multigene Panel Testing Among Young Breast Cancer Patients

**DOI:** 10.1007/s13187-019-01646-8

**Published:** 2019-12-04

**Authors:** T Jones, MS Trivedi, X Jiang, T Silverman, M Underhill, WK Chung, R Kukafka, KD Crew

**Affiliations:** 1 Florida Atlantic University, Christine E. Lynn College of Nursing, Boca Raton, FL 33431 USA; 2 Columbia University Irving Medical Center, New York, NY 10032 USA; 3 Dana Farber Cancer Institute, Boston, MA 02215 USA

**Keywords:** *BRCA1/2* genetic testing, Multigene panel testing, Young breast cancer patients, Racial/ethnic minorities

## Abstract

Genetic testing for hereditary breast and ovarian cancer (HBOC) is recommended for breast cancer patients diagnosed at age ≤ 50 years. Our objective was to examine racial/ethnic differences in genetic testing frequency and results among diverse breast cancer patients. A retrospective cohort study among women diagnosed with breast cancer at age ≤ 50 years from January 2007 to December 2017 at Columbia University in New York, NY. Among 1503 diverse young breast cancer patients, nearly half (46.2%) completed HBOC genetic testing. Genetic testing completion was associated with younger age, family history of breast cancer, and earlier stage, but not race/ethnicity or health insurance status. Blacks had the highest frequency of pathogenic/likely pathogenic (P/LP) variants (18.6%), and Hispanics and Asians had the most variants of uncertain significance (VUS), 19.0% and 21.9%, respectively. The percentage of women undergoing genetic testing increased over time from 15.3% in 2007 to a peak of 72.8% in 2015. Over the same time period, there was a significant increase in P/LP and VUS results. Due to uncertainty about the clinical implications of P/LP variants in moderate penetrance genes and VUSs, our findings underscore the need for targeted genetic counseling education, particularly among young minority breast cancer patients.

## Introduction

Germline genetic testing is the recommended standard of care for young breast cancer patients diagnosed at age ≤ 50 years due to an increased risk of hereditary breast and ovarian cancer (HBOC) syndrome [1]. *BRCA1* and *BRCA2* (*BRCA1/2*) gene mutations confer breast and ovarian cancers risks from 35% to 65% and 5% to 50%, respectively [2]. In addition, numerous other high-penetrance genes such as *CDH1*, *PTEN*, *STK11*, *TP53*, and *PALB2* are associated with hereditary breast cancer, making multigene panel testing for hereditary cancer risk of critical importance in young breast cancer patients [3]. Although multigene panel testing offers more comprehensive cancer risk assessment, there is greater uncertainty in clinical decision-making due to increased likelihood of variants of uncertain significance (VUS), particularly among racial/ethnic minorities who have been found to have more frequent VUS results compared to non-Hispanic Whites [4]. Once a pathogenic variant is identified, interventions available include risk-reducing contralateral prophylactic mastectomy, bilateral salpino-oophorectomy (BSO), or intensive screening with annual mammography and breast magnetic resonance imaging (MRI) for early detection [2, 5]. In addition, individuals with a *BRCA1/2* gene mutation with HER2-negative metastatic breast cancer may benefit from treatment with a PARP inhibitor. Young breast cancer patients diagnosed at 50 years or younger represent approximately 19% of new breast cancer cases in the USA [6]. In comparison to postmenopausal women, younger women are more likely to develop aggressive subtypes of breast cancer, have a worse prognosis with increased risk of recurrence, and have higher overall mortality [7]. Young breast cancer patients are also more likely to be diagnosed with triple-negative breast cancer (TNBC), which is associated with a higher frequency of *BRCA1* mutations [8]. Previous investigations among young breast cancer patients have found an underutilization of germline genetic testing [9, 10], but have been limited by small sample size, lack of diversity, and examination of *BRCA1/2* genes only. Larger studies of young breast cancer patients are needed to provide a comprehensive understanding of multigene panel testing. To address this gap, we examined frequency and predictors of *BRCA1/2* and multigene panel testing and assessed racial/ethnic differences in genetic testing frequency and results (pathogenic/likely pathogenic [P/LP], VUS, negative) among diverse women with early-onset breast cancer.

## Methods

We conducted a retrospective cohort study among women diagnosed with early-onset breast cancer (age ≤ 50 years) at Columbia University Irving Medical Center (CUIMC) in New York, NY from 2007 to 2017. Eligible participants included women diagnosed with invasive breast cancer or ductal carcinoma in situ (DCIS) at age ≤ 50 years between January 2007 and December 2017 with electronic health record (EHR) data available at CUIMC. Participants were ascertained via the New York-Presbyterian Hospital (NYPH) Tumor Registry at CUIMC. This study was approved by the Institutional Review Board at CUIMC and Florida Atlantic University. All participants met current National Comprehensive Cancer Network (NCCN) criteria for further cancer risk assessment evaluation for HBOC risk [1].

Patient demographics including age at diagnosis, race/ethnicity (non-Hispanic White, non-Hispanic Black, Hispanic, Asian, and other/unknown), marital status (married and unmarried), primary health insurance status (Medicare, Medicaid, private insurance, and other/uninsured), any structured family history of breast cancer (yes/no), breast tumor characteristics (i.e., stage at diagnosis), and year of diagnosis (prior to 2008, 2008–2010, 2011–2013, and after 2013) were collected through data extraction from the electronic health record (EHR) and New York-Presbyterian Hospital (NYPH) Tumor Registry.

Our primary outcome was the completion of germline genetic testing, dichotomized as genetic testing (yes/no) and classified by either *BRCA1/2* genetic testing only or multigene panel testing. Manual chart review was completed to extract genetic test results from the EHR, defined as positive (pathogenic/likely pathogenic [P/LP] variant), negative (no pathogenic variant), or variant of uncertain significance (VUS) at the time the original report was issued. We also extracted data on multigene panel testing (yes/no), the number of genes tested, and the frequency of P/LP variants or VUS for each gene.

## Statistical Analysis

We used descriptive statistics to characterize the study population, including demographic and clinical characteristics, and genetic testing frequency and results. Chi-squared test, or Fisher’s exact test for cell ranges below 5, was utilized to assess differences in study population characteristics among women who had genetic testing performed and those who did not. Two-sample *t* test was used to compare frequency distributions of continuous variables between those who completed and those who did not complete genetic testing, and the Satterthwaite approximation was used when variances were unequal. Univariate analysis was conducted to give an unadjusted estimate of the risk associated with each variable on frequency of genetic testing. We performed multivariate logistic regression analyses to examine the association between race/ethnicity and frequency of genetic testing, while controlling for covariates. Variables were included in the multivariate model if they were significant (*p* < 0.20) in the univariate model and if they changed the parameter estimate by at least 10%. A secondary analysis of participants who completed genetic testing was also conducted using the same methodology. The subset of participants who completed genetic testing was stratified by genetic test results (P/LP variant, VUS, negative). To test for changes in genetic testing over time, we used the Cochran-Armitage test for trend. All statistical analysis was conducted using SAS version 9.4 (SAS Institute, Cary, NC), and a *p* value < 0.05 was considered statistically significant.

## Results

**Fig. 1:**
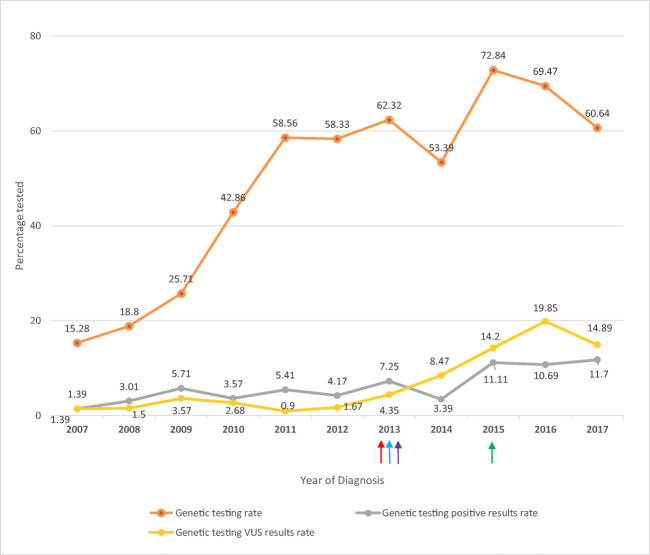
Trends over time in germline genetic testing, positive and VUS results in a cohort of young women diagnosed with breast cancer (*N* = 607) by year of diagnosis at Columbia University Irving Medical Center, New York, NY (2007–2017). Red arrow indicates when the Affordable Care Act clarified coverage for *BRCA1/2* genetic testing to guarantee zero out-of-pocket costs, 2013. Blue arrow indicates when actor Angelina Jolie disclosed her *BRCA* genetic testing results, May 2013. Purple arrow indicates the US Supreme court vs. Myriad case, which increased the number of labs offering testing, 2013. Green arrow indicates the American College of Medical Genetics and Genomics (ACMG) Standards and guidelines for the interpretation of sequence variants, 2015

A total of 1621 patients with breast cancer diagnosed at age 50 years or younger between 2007 and 2017 at CUIMC were identified, and 118 patients were excluded from the study, including 94 (5.8%) with lobular carcinoma in situ, 19 (1.2%) with no available EHR data at CUIMC, and 5 (0.3%) males. Among 1503 evaluable patients, mean age of 42.7 years (SD, 5.8), 42.4% were non-Hispanic White, 13.3% non-Hispanic Black, 25.5% Hispanic, 9.9% Asian, and 8.9% other or unknown race/ethnicity. The majority (60.5%) of patients had private insurance, 22.8% had Medicaid, 7.2% had Medicare (either due to disability or existing co-morbidities), and 9.4% had other insurance or were uninsured. Nearly half (46.2%) completed genetic testing (Table 1). Participants who completed genetic testing were more likely to be younger, be married, have a family history of breast cancer, have stage 1 breast cancer, and be diagnosed after 2013. There were no significant differences in the completion of genetic testing based upon race/ethnicity or primary health insurance status. Among 683 women who completed genetic testing (Table 2), 12.7% had P/LP variants and 13.9% had VUSs. Among 87 P/LP variants detected, 46.0% were in *BRCA1*, 27.6% *BRCA2*, 6.9% *CHEK2*, 4.6% *ATM*, and 14.9% in nine other genes. Among 130 VUSs detected, 11.5% were in *BRCA1*, 10.8% *BRCA2*, 10.0% *ATM*, 6.9% *CHEK2*, and 60.8% in 32 other genes. Non-Hispanic Blacks and Whites had the highest frequency of P/LP variants (18.2% and 16.3%, respectively), whereas Asians and Hispanics had the highest frequency of VUSs (21.9% and 19.0%, respectively). The percentage of women undergoing HBOC genetic testing increased over time (Cochran-Armitage test for trend, *p* < .001), from 15.3% in 2007 to a peak of 72.8% in 2015 (Fig. 1). Over the same time period, there was a significant increase in P/LP and VUS results.

**Table 1:** Baseline characteristics stratified by genetic testing completion and multivariable regression model of the association between sociodemographic and clinical factors and genetic testing completion among young breast cancer patients diagnosed at Columbia University Irving Medical Center, New York, NY (2007–2017)

Characteristic	No genetic testing *N* = 808 (53.8%)	Genetic testing *N* = 695 (46.2%)	*p* value	Multi-variable*; odds ratio	95% confidence interval	*p* value
Mean age at diagnosis, years (SD)	43.6 (5.5)	41.7 (6.0)	*< .001*	0.93	(0.91, 0.95)	*< .001*
Race/ethnicity						
Non-Hispanic White	355 (43.9)	282 (40.6)	0.242	1.00		
Non-Hispanic Black	110 (13.6)	90 (12.9)		1.01	(0.66, 1.48)	0.969
Hispanic	191 (23.6)	192 (27.6)		1.36	(0.98, 1.89)	0.068
Asian	75 (9.3)	74 (10.6)		0.89	(0.59, 1.33)	0.558
Other	77 (9.5)	57 (8.2)		1.02	(0.65, 1.60)	0.939
Marital status						
Married	326 (40.3)	304 (43.7)	0.132	1.32	(1.03, 1.71)	*0.030*
Unmarried	482 (59.7)	391 (56.3)		1.00		
Insurance						
Private	483 (59.8)	427 (61.4)	0.410	1.00		
Medicare	66 (8.2)	42 (6.0)		1.25	(0.75, 2.06)	0.391
Medicaid	180 (22.3)	163 (23.5)		0.82	(0.59, 1.12)	0.210
Uninsured/other	79 (9.8)	63 (9.1)		0.78	(0.51, 1.20)	0.260
Stage						
0	139 (17.2)	95 (13.7)	*< .001*	0.62	(0.43, 0.88)	*0.007*
1	219 (27.1)	262 (37.7)		1.00		
2	194 (24.0)	220 (31.7)		0.91	(0.67, 1.23)	0.538
3	72 (8.9)	58 (8.3)		0.84	(0.54, 1.32)	0.452
4	55 (6.8)	36 (5.2)		0.38	(0.23, 0.65)	*< .001*
Unknown	129 (16.0)	24 (3.5)		0.37	(0.22, 0.63)	*< .001*
Family history of breast cancer						
Yes	96 (11.9)	203 (29.2)	*< .001*	2.84	(2.09, 3.86)	*< .001*
No	712 (88.1)	492 (70.8)		1.00		
Year of diagnosis						
Prior to 2008	316 (39.1)	61 (8.8)	*< .001*	1.00		
2008–2010	168 (20.8)	84 (12.1)		2.78	(1.86, 4.17)	*0.004*
2011–2013	148 (18.3)	221 (31.8)		8.34	(5.73, 12.13)	*< .001*
After 2013	176 (21.8)	329 (47.3)		10.29	(7.09, 14.94)	*< .001*

Entries in italics indicate statisticially significant variables

*Multivariable logistic regression model was adjusted for age, insurance, stage of breast cancer, family history of breast cancer, and year of diagnosis

**Table 2:** Patient characteristics by genetic test results among young women diagnosed with breast cancer at Columbia University Irving Medical Center, New York, NY (2007–2017)

Characteristic	Genetic test result: Positive *N* (%)^1^ *N* = 87 (12.7%)	Genetic test result: VUS N (%)^1^ *N* = 95 (13.9%)	Genetic test result: Negative *N* (%)^1^ *N* = 501 (73.4%)	Total *N* (%)^2^ *N* = 683^3^	*p* value
Age at diagnosis (years)					
Mean (SD)	40.6 (6.5)	41.4 (6.3)	41.9 (5.8)	41.7 (6.0)	0.150
Race/ethnicity, *N* (%)					
Non-Hispanic White	45 (16.3)	24 (8.7)	207 (75.0)	276 (40.4)	
Non-Hispanic Black	16 (18.2)	12 (13.6)	60 (68.2)	88 (12.9)	
Hispanic	15 (7.9)	36 (19.0)	138 (73.0)	189 (27.7)	
Asian	6 (8.2)	16 (21.9)	51 (69.9)	73 (10.7)	
Other	5 (8.8)	7 (12.3)	45 (78.9)	57 (8.3)	*0.003*
Marital status, *N* (%)					
Married	34 (11.4)	33 (11.1)	231 (77.5)	298 (43.6)	
Unmarried	53 (13.8)	62 (16.1)	270 (70.1)	385 (56.4)	0.091
Insurance, *N* (%)					
Private	55 (13.1)	57 (13.6)	307 (73.3)	419 (61.3)	
Medicare	3 (7.1)	3 (7.1)	36 (85.7)	42 (6.1)	
Medicaid	21 (13.0)	19 (11.8)	121 (75.2)	161 (23.6)	
Uninsured/other	8 (13.1)	16 (26.2)	37 (60.7)	61 (8.9)	*0.037*
Stage, *N* (%)					
0	7 (7.6)	7 (7.6)	78 (84.8)	92 (13.5)	
1	36 (14.0)	33 (12.8)	189 (73.3)	258 (37.8)	
2	30 (13.9)	39 (18.1)	147 (68.1)	216 (31.6)	
3	7 (12.1)	8 (13.8)	43 (74.1)	58 (8.5)	
4	3 (8.6)	5 (14.3)	27 (77.1)	35 (5.1)	
Unknown	4 (16.7)	3 (12.5)	17 (70.8)	24 (3.5)	0.342
Family history of breast cancer, *N* (%)					
Yes	31 (15.4)	28 (13.9)	142 (70.6)	201 (29.4)	
No	56 (11.6)	67 (13.9)	359 (74.5)	482 (70.6)	0.344
Year of diagnosis, *N* (%)					
Prior to 2008	6 (9.8)	5 (8.2)	50 (82.0)	61 (8.9)	
2008–2010	13 (15.9)	8 (9.8)	61 (74.4)	82 (12.0)	
2011–2013	21 (9.6)	9 (4.1)	188 (86.2)	218 (31.9)	
After 2013	47 (14.6)	72 (22.7)	202 (62.7)	322 (47.1)	*< .001*

Entries in italics indicate statisticially significant variables

^1^ Row percentages

^2^ Column percentages

^3^ Excludes 12 patients- had genetic testing but no documentation of the results in their medical record

In multivariable analysis (Table 1), women who were older at breast cancer diagnosis were less likely to have genetic testing (odds ratio [OR], 0.93; 95% confidence interval [CI], 0.91–0.95; *p* < 0.001). The odds of a young woman completing genetic testing increased nearly 3-fold if she had a family history of breast cancer (OR, 2.84; 95% CI, 2.09–3.86; *p* < 0.001). Compared to patients with stage 1 breast cancer, those with stage 0 or stage 4 disease at diagnosis were less likely to complete genetic testing (OR, 0.62; 95% CI, 0.43–0.88; *p* = 0.007 and OR, 0.38; 95% CI, 0.23–0.65; *p* < 0.001, respectively). Compared to those diagnosed before 2008, patients diagnosed after 2013 were over 10 times more likely to have genetic testing (OR, 10.29; 95% CI, 7.09–14.94; *p* < 0.001).

## Discussion

We examined frequency and predictors of completion of *BRCA1/2* and multigene panel testing and assessed racial/ethnic differences in genetic testing frequency and results among breast cancer patients diagnosed at age ≤ 50 years. Nearly half of the young breast cancer patients completed genetic testing with no differences found in frequency based on race/ethnicity. In our multivariable analysis, we found that younger age at diagnosis, family history of breast cancer, stage of diagnosis, and year of diagnosis were important predictors of the completion of genetic testing. Similar to recent findings [11], we found that the percentage of young women diagnosed with breast cancer who completed germline genetic testing increased over time, with 73% of patients completing HBOC genetic testing in 2015.

Few studies in the USA have addressed germline genetic testing in young diverse breast cancer patients in an era of multigene panel testing. Two previous studies that examined the frequency of *BRCA1/2* genetic testing in young breast cancer patients diagnosed at age ≤ 45 years found that 72.9% of their sample [12, 13] had genetic testing in one study [12], and 87% of the women reported testing 1-year post diagnosis in the second study [14]. Other previous studies of young women with breast cancer found lower frequency of *BRCA1/2* genetic testing among young breast cancer patients ranging from 21% to 24% reporting that they completed testing, but these studies were based on self-report and limited to *BRCA1/2* genetic testing only [9, 15]. Our results extend the current literature as we also examined completion of multigene panel testing among diverse young breast cancer patients.

Our findings are consistent with previous studies showing that women with a family history of breast cancer and those diagnosed in recent years were more likely to have genetic testing [12, 14]. This suggests an increased awareness and improvement in access to cancer genetic testing based on national recommendations. However, it is important to consider factors that have impacted the increase in genetic testing over the years. In 2013, the Affordable Care Act (ACA) provided coverage for *BRCA1/2* genetic testing to guarantee zero out-of-pocket costs, which may have increased accessibility of genetic testing [16]. In a review exploring the impact of the “Angelina Jolie Effect,” Gianmarco et al. reported an increase of referrals for genetic counseling with a nearly 3-fold increase in *BRCA1/2* genetic testing [17]. Additionally, in 2013 in the case of Association for Molecular Pathology v. Myriad Genetics, the US Supreme Court ruled unanimously that naturally occurring DNA was a product of nature and not patent eligible [18]. Rising public awareness about *BRCA1/2* genetic testing since 2013 has also led to an increase in receipt of genetic testing [19]. Recent recommendations from the American College of Medical Genetics and Genomics (ACMG) specify the standards for the interpretations of sequence variations, which possibly increased the frequency of VUS, as variant calling became more common [20].

In our study, we found that women with metastatic breast cancer were over 60% less likely to undergo genetic testing compared to women with stage I disease, which is consistent with the prior literature [21]. Recently, *BRCA1/2* genetic test results have become increasingly relevant for systemic treatment decisions with the use of poly ADP-ribose polymerase (PARP) inhibitors for *BRCA1/2*-associated metastatic breast cancer [22]. Furthermore, once a cancer-predisposing P/LP variant has been identified in patients with breast cancer, at-risk relatives can also receive genetic testing.

This study provides insights into P/LP variants in HBOC genes among young breast cancer patients. Furthermore, we observed racial/ethnic disparities in genetic testing results with the highest frequency of P/LP variants among young non-Hispanic Black breast cancer patients followed by non-Hispanic women of European ancestry. One possible explanation is that Black breast cancer patients with extensive family history and strong breast cancer risk factors elect to have genetic testing. Since the burden of breast cancer is particularly high among young Black women, with a mortality rate that is two times greater among young women of European ancestry [23], there is a need to engage more young Black breast cancer patients in genetic counseling education and the importance of having HBOC genetic testing performed. In addition, the frequency of VUSs was highest among Asians and Hispanics compared to the other racial/ethnic groups in our sample. These findings corroborate recent studies that have also reported higher frequencies of VUS among racial/ethnic minority breast cancer patients compared to women of European ancestry due to limited understanding of the normal spectrum of genetic variation in minority groups [4, 11]. Since ethnic minority breast cancer patients may have lower health literacy, increased distress with VUS results, and confusion about the implications of VUSs, our findings underscore the need for targeted genetic counseling education among minority women [24]. However, the overwhelming majority of VUS will be reclassified as likely benign/benign over time with increasing data on allele frequencies across all ethnic groups, increasing data deposition to databases such as ClinVar, improved prediction algorithms, and functional studies [25].

Strengths of our study include a large sample size of women with early-onset breast cancer diagnosed over a long time period, a racially/ethnically diverse sample, and detailed clinical data from the EHR. Limitations include the retrospective cohort design and under-reporting of genetic testing, if counseling and testing occurred at an outside institution. Since this study was conducted at a single urban academic institution with access to genetic counseling services, our results may not be generalizable to other populations and should be confirmed in other geographic and clinical settings.

In summary, we did not observe a difference in HBOC genetic testing among young breast cancer patients based upon race/ethnicity; however, we observed a high frequency of pathogenic/likely pathogenic variants among non-Hispanic Black and non-Hispanic White women and there were more VUS results among minority women. Due to uncertainty about the clinical implications of P/LP variants in moderate penetrance genes and VUSs, our findings underscore the need for targeted genetic counseling education, particularly among young minority women [24].
